# Spectral Reconstruction from RGB Imagery: A Potential Option for Infinite Spectral Data?

**DOI:** 10.3390/s24113666

**Published:** 2024-06-05

**Authors:** Abdelhamid N. Fsian, Jean-Baptiste Thomas, Jon Y. Hardeberg, Pierre Gouton

**Affiliations:** 1 Imagerie et Vision Artificielle (ImVIA) Laboratory, Department Informatique, Electronique, Mécanique (IEM), Université de Bourgogne, 21000 Dijon, France; jean-baptiste.thomas@u-bourgogne.fr (J.-B.T.); pgouton@u-bourgogne.fr (P.G.); 2 Colourlab, Department of Computer Science, Norwegian University of Science and Technology (NTNU), 2815 Gjøvik, Norway; jon.hardeberg@ntnu.no

**Keywords:** spectral imaging, spectral reconstruction, RGB imagery

## Abstract

Spectral imaging has revolutionisedvarious fields by capturing detailed spatial and spectral information. However, its high cost and complexity limit the acquisition of a large amount of data to generalise processes and methods, thus limiting widespread adoption. To overcome this issue, a body of the literature investigates how to reconstruct spectral information from RGB images, with recent methods reaching a fairly low error of reconstruction, as demonstrated in the recent literature. This article explores the modification of information in the case of RGB-to-spectral reconstruction beyond reconstruction metrics, with a focus on assessing the accuracy of the reconstruction process and its ability to replicate full spectral information. In addition to this, we conduct a colorimetric relighting analysis based on the reconstructed spectra. We investigate the information representation by principal component analysis and demonstrate that, while the reconstruction error of the state-of-the-art reconstruction method is low, the nature of the reconstructed information is different. While it appears that the use in colour imaging comes with very good performance to handle illumination, the distribution of information difference between the measured and estimated spectra suggests that caution should be exercised before generalising the use of this approach.

## 1. Introduction

Spectral imaging systems (SIs) capture the distribution of light in a scene across several spectral bands. As a result, they offer more complete visual data compared to conventional colour cameras, which only operate within three broad spectral bands (red, green, and blue). SIs present numerous advantages for various computer vision applications such as medical imaging [1,2], remote sensing [3,4], and object tracking [5], to cite a few. Nonetheless, their utilisation has been constrained by factors such as size, cost, and low spatial resolution. Notably, the limitations stem from the availability and diversity of spectral data.

The computer vision field evolved together with imaging technology from grayscale to colour, and from colour to multi-modal, spectral, or polarisation imaging. Each evolution is bringing access to new information in order to overcome the limitations of the previous modality. Despite remarkable performance with colour images, spectral imaging emerges as a promising avenue specifically tailored to address the limitations of colour imaging, offering a richer and more nuanced understanding of the underlying data [6]. This shift in emphasis underscores the need to explore beyond conventional RGB datasets and highlights spectral imaging as a key approach in overcoming the constraints associated with colour representation.

Existing spectral databases have played a pivotal role in advancing research in computer vision, providing valuable datasets for various applications [7]. However, the current repositories, though valuable, face limitations in terms of diversity, scale, and representation of real-world scenarios. These databases often cover specific domains or scenes, making them less suitable for broader applications. It is worth mentioning that existing databases cannot be concatenated, mostly because of standardisation problems, spectral specificity, and spatial resolution [8].

**Figure 1:**
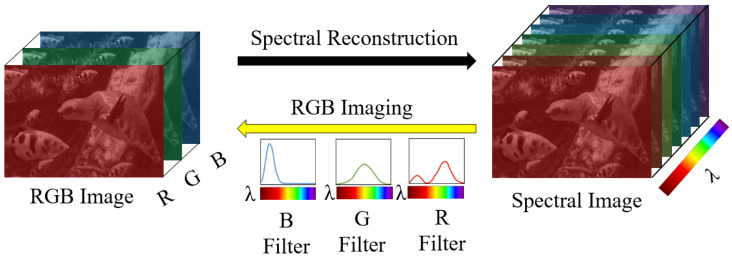
Spectral reconstruction from an RGB image, where the spectral reconstruction model estimates the original spectral information from the RGB image.

Furthermore, a richer and more diverse spectral database would enable researchers to explore a wider range of applications beyond the current scope. These include, but are not limited to, fields such as object detection, scene understanding, and autonomous driving, where spectral imaging holds immense potential. Notably, these tasks often demand robust deep learning models, necessitating a substantial volume of high-quality training data. Through spectral reconstruction (SR) (Figure 1), also referred to as spectral uplifting or spectral superresolution, we gain access to a practically unlimited source of RGB images present in various datasets like Imagenet [9].

This article explores the limitation of reconstructed spectral data from RGB by conducting experiments over the spectral distribution and colorimetric relighting analysis. Three primary initiatives have been considered for obtaining spectral data. The first involves capturing more data using spectral cameras. However, this approach faces challenges related to standardisation, given the varying configurations of spectral cameras [8]. The second method entails generating data in computer graphics [10], leveraging tools like Mitsuba [11], but is often tied to specific reflectance models, and the low number of scenes created lacks generalisation ability. Lastly, the third initiative, which forms the focus of this study, revolves around spectral reconstruction from RGB data [7]. This cutting-edge technique marks a significant advancement in the field, as it enables the transformation of conventional RGB images into highly detailed representations encompassing a more extensive range of spectral information.

This article is structured as follows. In Section 2, we delve into the existing body of work related to spectral reconstruction methods. In Section 3, we present our experimental protocol, discussing both the spectral dataset and the deep learning-based model used, as well as the application of performance-based metrics, along with spectral analysis using principal component analysis (PCA). Section 4 serves as the analysis section, where we delve into the interpretation and commentary on the obtained results. In addition to the analysis of reconstruction accuracy and spectral information representation, Section 4 also includes a colorimetric analysis. Specifically, we compute the Euclidean distance $\Delta E_{ab}^{*}$ between the spectral reconstructed data and the ground-truth spectral data under various illuminants, providing further insights on how the spectral reconstruction quality impacts the specific colour imaging application. Finally, Section 5 presents our conclusion, summarising key insights and implications and proposing potential avenues for future research. In this context, we show, on two independent datasets and two different spectral reconstruction methods, that the information in the original spectra and the estimated ones seems very different on a PCA space, which suggest caution in the use of the estimated data. On the other hand, we also show that, from a colorimetric perspective, the estimated spectra are sufficient to perform relighting of the scene or chromatic adaptation.

## 2. Related Work

### 2.1. Spectral Image Acquisition

Recent advancements in imaging systems have introduced various sophisticated techniques for capturing spectral images. Despite the progress, these methods still face significant challenges. Traditional scanning techniques, although widely used, are often slow and cumbersome. Technologies such as pushbroom and whiskbroom scanners, commonly employed in remote sensing and other applications, require time-consuming processes and large, non-portable equipment [12,13].

In an effort to address these limitations, innovative solutions like snapshot compressive imaging (SCI) systems have been developed. These systems can compress complex hyperspectral data into a single 2D image, offering a more efficient approach compared to conventional methods [12,14,15,16,17]. Among these, the Coded Aperture Snapshot Spectral Imaging (CASSI) system stands out for its potential to revolutionise the field [16,18].

However, despite their potential, these advanced imaging systems are still limited by high costs and practical challenges. The expense of SCI systems makes them inaccessible for broader use. Additionally, issues such as spectral estimation errors persist, impacting the accuracy and reliability of the captured data [19].

These challenges highlight the critical need for further research in spectral reconstruction. Improving these technologies to be more affordable, efficient, and accurate is essential for their widespread adoption and application in various fields.

### 2.2. SR from RGB

The first SR techniques looked for three-dimensional linear spectrum models. It was then demonstrated that the spectra may be precisely retrieved from RGB using a linear transform if such a “3D” linear model is applicable [20,21]. Simple statistical models like regression [22,23,24] and Bayesian inference [25,26] have been provided, which facilitate higher- or full-dimensional spectrum recovery, despite the fact that a 3D model can only cover a limited variance in real-world spectra [27,28]. With the growing quantity of accessible data, novel methods such as deep neural networks (DNN) [29,30,31,32,33,34], sparse coding [35], and shallow networks [36,37,38] have been based on richer inference algorithms. A comprehensive comparison of the approaches is not yet accessible, though, because not all early and modern methods have been benchmarked on the same database. Nonetheless, it is reasonable to state that DNNs are acknowledged as the top SR technique.

Regression [22], one of the earliest techniques, has become popular because of its straightforward, fast, accurate, and closed-form solution. RGB and their spectral estimations are related in the most basic “linear regression” [22] by a single linear transformation matrix. Moreover, polynomial and root-polynomial regression [23,24] expand the RGB into polynomial/root-polynomial terms, which are subsequently transferred to spectra using a linear transform, in order to add non-linearity. Regressions that minimise the mean squared error (MSE) in the training set are sometimes referred to as “least-squares” regressions. Nevertheless, Lin and Finlayson [39] proposed a “relative-error-least-squares” minimisation strategy for regressions, which further enhances the performance of regression-based SR because SRs are—at least recently—more frequently assessed using relative errors [20,29,35,40].

Many recent methods for SR rely on DNN architectures, specifically, convolutional neural networks (CNNs) or generative adversarial networks (GANs), where large image patches serve as standard inputs. In the NTIRE 2018, 2020, and 2022 Spectral Reconstruction Challenges, DNN-based solutions dominated the top rankings. For instance, the NTIRE 2018 challenge was won by “HSCNND”, which utilised a densely connected structure, while “AWAN” emerged victorious in the NTIRE 2020 challenge, employing an attention network structure. Notably, the latest winner of the NTIRE challenge (edition 2022), “MST++”, proposed a novel approach using a transformer-based model for efficient spectral reconstruction. However, despite these advancements, most DNN evaluations are conducted on optimally captured images, neglecting more challenging real-world conditions such as exposure variations and diverse scene compositions. Comprehensive assessments reveal that DNNs are often susceptible to exposure changes, unfamiliar scenes, and scenes lacking specific image contents [41].

Initially, spectral reconstruction relied on regression-based [42] and sparse-coding techniques [43]. While not completely replacing linear methods, deep learning models [33], which are mostly non-linear procedures, have considerably increased in popularity in recent years. Moreover, community challenges have recently emerged to stimulate research in developing robust and reliable networks for spectral reconstruction from RGB images [7,35]. Consequently, spectral reconstruction techniques have been extensively explored within the research community, with a multitude of studies contributing to this area [35,43]. For a detailed overview and in-depth information, we direct the interested reader to the comprehensive review on spectral reconstruction methods in the literature [44]. Spectral reconstruction from RGB images has been significantly influenced by the pioneering work of the colour imaging community, as demonstrated by [45]. In the image formation model, the spectral function r(λ) represents the intensity distribution across wavelengths that is defined as the radiance spectrum. In accordance with this, the sensitivities of the R, G, and B sensors are represented as $s_{k}\left(\lambda \right)$, where k=R,G,B. Therefore, the RGB image creation is expressed as the inner productbetween the spectral sensitivity and the measured radiance [45]:

(1) $$\rho_{k}=\sum_{\lambda \in \omega }s_{k}\left(\lambda \right)r\left(\lambda \right)$$

where ω denotes the visible range, which in this article is set to [400, 700] nanometers, and λ∈ω. Moreover, the ground-truth spectra are sampled at n equally spaced wavelengths. Equation (1) can therefore be vectorised:

(2) $$S^{T}\underline{r}=\underline{\rho }$$

where the 3-value RGB colour is represented by $\rho ={\left(R,G,B\right)}^{T}$, the n×3 spectral sensitivity matrix is $S=({\underline{s}}_{R},{\underline{s}}_{G},{\underline{s}}_{B})$, and $r\in R_{n}$ is the discrete representation of spectra. The linear colour or raw camera response, which is frequently utilised as ground-truth RGB for training spectral reconstruction algorithms [29], is denoted by this ρ vector.

Spectral reconstruction methods are employed to map RGB colours to spectral estimations. This mapping is accomplished through a representation of the spectral reconstruction algorithm using a specific mapping function $\psi :R^{3}\Rightarrow R^{n}$; SR can be written as follows:

(3) $$\psi \left(\underline{\rho }\right)\approx \underline{r}$$

## 3. Methodology

Given the maturation of spectral reconstruction methodologies, we advocate for the adoption of state-of-the-art approach for deriving spectral information from RGB imagery, but we want to emphasise the limitation of the technique. Therefore, to validate the generalisation capability and replicability of the MST++ [46] and A++ [47] models in spectral reconstruction, and to demonstrate their potential applicability to diverse scenarios, we assess their performance on unseen data. Additionally, our investigation extended to analysing the spectral distribution patterns using the Spectral Image Database for Quality dataset (SIDQ) [48], which introduced a hyperspectral image database consisting of nine scenes. These scenes were meticulously chosen to represent diverse materials such as textile, wood, and skin. The dataset provides spectral reflectance data, acquired using a hyperspectral system (HySpex VNIR-1600 manufactured by Neo, Oslo, Norway), with a spectral range spanning from 410 to 1000 nm, containing 160 spectral bands (where 85 bands are in the visible light spectrum), coded over 16 bits. Importantly, the SIDQ dataset includes not only hyperspectral data but also their RGB counterparts. To ensure comparability of the results obtained from the different models and datasets used in this study, we unified the interval by considering only the bands from 410 to 700 nm when working with the SIDQ dataset. As we possess the RGB counterparts of the hyperspectral images, our next step involves leveraging SR models to reconstruct spectra from the RGB images. It is essential to note that the resulting spectra will be in the interval of [400, 700] nm. Subsequently, we compare the reconstructed spectra obtained through the MST++ and A++ models with the original hyperspectral images from the SIDQ dataset. This comparative analysis provides insights into the accuracy and efficacy of the spectral reconstruction model, offering a robust evaluation of our approach. Moreover, we used the CAVE dataset [49] in addition to the SIDQ dataset for our analysis. The CAVE dataset contains 32 different spectral reflectance data spanning from 400 to 700 nm, with 31 spectral bands coded over 16 bits and their RGB counterparts. Both the CAVE and SIDQ datasets are normalised between 0 and 1. This normalisation, along with the diversity in terms of the number of scenes and spectral bands, allows for a more comprehensive evaluation and generalisation capability of the models used in this study.

### Evaluation Metrics

To assess the accuracy and fidelity of the spectral reconstruction, we employed quantitative performance metrics, including Peak Signal-to-Noise Ratio (PSNR), Structural Similarity Index (SSIM), and Mean Relative Absolute Error (MRAE), computed between the spectral reconstructed image $\hat{x}$ and the ground-truth images *x*. These metrics provide a comprehensive evaluation of the reconstructed spectra by quantifying the similarity and deviation from the ground truth spectral data.

1. Root Mean Square Error:
  (4) $$RMSE=\sqrt{\frac{1}{n}{∥x-\hat{x}∥}_{2}}$$
  where *n* represents the number of spectral bands. Moreover, RMSE is scale-dependent, that is, the overall brightness level in which the compared spectra reside will reflect on the scale of RMSE;
2. Peak Signal-to-Noise Ratio:
  (5) $$PSNR=20\times \log_{10}\left(\frac{x_{\max}}{RMSE}\right)$$
  where $x_{\max}$ is the maximum possible value for our images;
3. Structural Similarity Index:
  (6) $$\mathrm{SSIM}\left(x,\hat{x}\right)=\frac{\left(2\mu_{x}\mu_{\hat{x}}+C_{1}\right)\left(2\sigma_{x\hat{x}}+C_{2}\right)}{\left(\mu_{x}^{2}+\mu_{\hat{x}}^{2}+C_{1}\right)\left(\sigma_{x}^{2}+\sigma_{\hat{x}}^{2}+C_{2}\right)}$$
  where $\mu_{x}$, $\mu_{\hat{x}}$, $\sigma_{x}^{2}$, and $\sigma_{\hat{x}}^{2}$ are the mean and variance of the reference image *x* and estimated image $\hat{x}$, respectively, while $\sigma_{x\hat{x}}$ is the covariance. The SSIM of all bands is acquired by calculating the SSIM of each channel separately and averaging all SSIMs;
4. Mean Relative Absolute Error:
  (7) $$MRAE=100\times \frac{1}{n}\left|\right|\frac{x-\hat{x}}{x}{\left|\right|}_{1}$$
  where *n* is the number of spectral channels, and we perform an element-wise division to compute the $L_{1}$ norm. In essence, the MRAE metric calculates the average absolute deviation across all spectral channels. This metric is widely recognised as the standard measure for ranking and assessing SR algorithms in the latest benchmark studies [29];
5. Entropy Similarity Metric:
  While commonly employed in fields like molecular spectroscopy [50] for its ability to capture the similarity in entropy distributions between spectra, entropy similarity remains relatively underutilised within the spectral imaging community. Unlike conventional metrics, Entropy Similarity provides a comprehensive assessment of the fidelity of spectral reconstruction by quantifying the agreement between the spectral entropy patterns of the reconstructed and ground truth spectra.
  (8) $$\mathrm{ES}\left(x,\hat{x}\right)=1-\frac{\left|H\left(x\right)-H\left(\hat{x}\right)\right|}{\max(H\left(x\right),H\left(\hat{x}\right))}$$
  where H(x) represents the entropy of image x. Similarly to SSIM and PSNR, ES is acquired by calculating the ES for each spectral channel separately and averaging all ESs. Since it is a similarity metric, a higher score indicates better alignment, with a score of 1 representing perfect alignment.

In addition to the performance metrics, we conducted PCA to examine the the variance in spectral distribution:

1. This analysis involved concatenating the reconstructed spectra (from both MST++ and A++) with the ground-truth spectral image. The concatenated data facilitated the generation of clouds of points, enabling a visual comparison of the spectral distributions. By aligning the reconstructed and original spectral data on the same axis, PCA allowed for a comprehensive exploration of the variance within the spectra;
2. Also, we performed PCA without concatenation, directly computing the eigenvectors to investigate the spectral distribution of reconstructed data, generated by both models, against original spectral data and their RGB counterpart. This approach provided insights into the underlying structures of the spectral data without the influence of concatenation. Through the computation of eigenvectors, we gained a deeper understanding of the spectral variability and the principal components driving the variance within the spectra.

**Figure 2:**
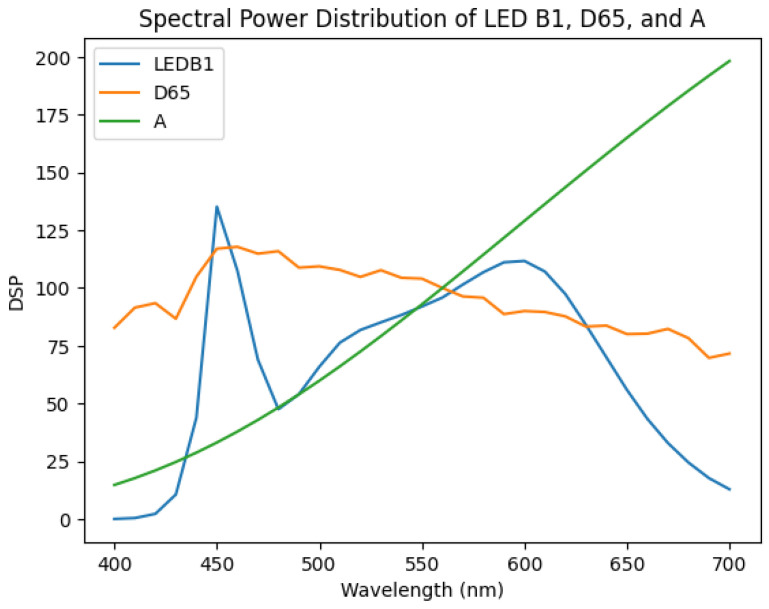
Spectral power distribution of the used illuminants.

**Figure 3:**
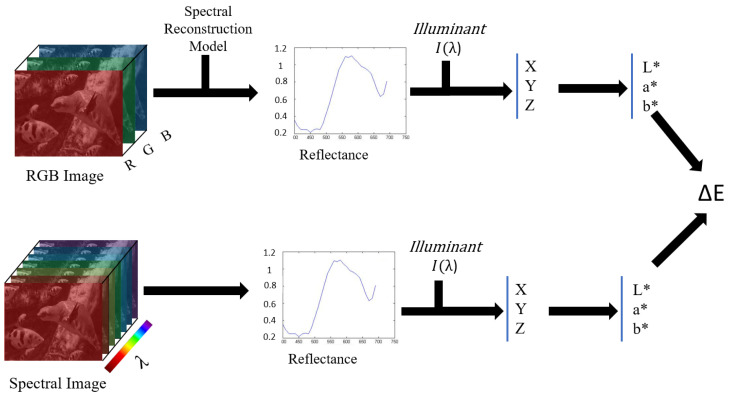
Colorimetric analysis between spectral reconstructed data and the original spectral image.

Furthermore, a relighting analysis was conducted to evaluate the colorimetric performance of the two state-of-the-art SR methods to observe their capacity to predict colorimetric values under different lights. We specifically considered Illuminants D65 and A, but also a white LED light, LED-B1 (see Figure 2). The analysis involved using reflectance factors from spectral data and multiplying them with the respective illuminant to obtain radiance data. For the reconstructed spectra (from both the A++ and MST++ models), we assumed an E illumination for the initial RGB images, implying that the colour images were white-balanced, thus approximating a flat spectral distribution. This step ensures that the reconstructed data maintain consistency with the assumed illumination conditions. It is noteworthy that such an assumption is not needed for the original spectral data provided by the CAVE and SIDQ datasets, as they are already provided as reflectance data. The radiance data were then converted to the CIE 1931 XYZ colour space using the 2 degrees standard observer colour-matching functions. Subsequently, the XYZ values were transformed into the CIELAB colour space to enable perceptually uniform colour comparisons [51]. Finally, the Euclidean distance was computed between the reconstructed spectral data and the ground-truth spectral data in the CIELAB colour space to assess the colour accuracy of the SR methods under different illumination conditions (see Figure 3). This colorimetric analysis provides valuable insights into the robustness and generalisation capabilities of the SR methods across varying lighting scenarios.

## 4. Analysis

### 4.1. Spectral Analysis

**Figure 4:**
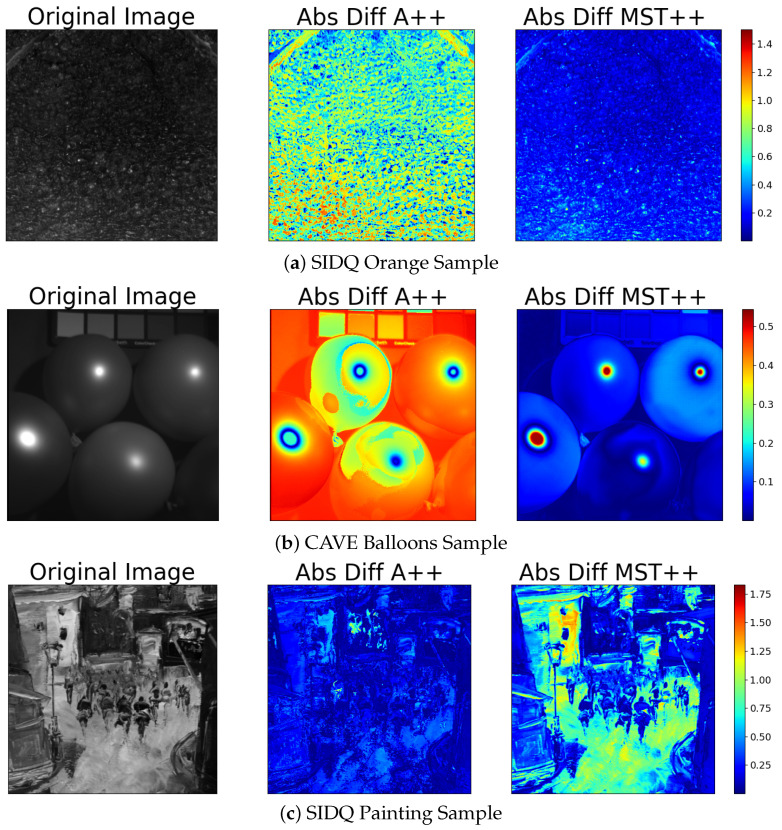
(**Left**): Original spectral band at 410 nm. (**Middle**): Heat map using absolute difference between the reconstructed spectral image from A++ model and the original spectral image. (**Right**): Heat map using absolute difference between the reconstructed spectral image from MST++ and the original spectral image.

Table 1 provides an overview of the performance metrics associated with spectral reconstruction models, specifically MST++ and A++, applied to the SIDQ dataset [48]. The metrics include PSNR, SSIM, MRAE, and ES. These metrics offer insights into the quality and fidelity of the reconstructed spectral data across different scenes within the SIDQ dataset. Moreover, Table 2 presents the same performance metrics (PSNR, SSIM, MRAE, and ES) for spectral reconstruction achieved by both the MST++ and A++ models across all scenes within the CAVE dataset [49]. It is important to note that neither MST++ nor A++ models were trained on both datasets. We observe that, for conventional metrics such as PSNR, SSIM, and MRAE, the transformer solution (MST++) outperforms the pixel-based solution (A++ model). While both models perform well, the A++ model outperforms MST++ for the Entropy Similarity (ES) metric, highlighting the importance of exercising caution when evaluating models. In addition, the illustrations in Figure 4 corroborate the findings in Table 1 and Table 2, revealing a notably low error map between the reconstructed spectral image from RGB and the original hyperspectral image across various scenes.

It is important to note that both models performed equivalently across the two datasets tested. However, there was a magnitude difference observed between the results obtained from the two datasets (see Table 1 and Table 2). We observed an overall better performance of the models when using the SIDQ dataset compared to the CAVE dataset. This observation can be attributed to the differences in scene content between the two datasets. Specifically, the CAVE dataset consists of more complex scenes with a higher prevalence of specular and dark areas, which can pose challenges for spectral image reconstruction algorithms. In contrast, the SIDQ dataset is characterised by smoother and flatter scenes with fewer specular and dark regions, which may facilitate more accurate reconstruction of spectral images. Therefore, the differences in scene complexity and the presence of specular and dark areas could explain the observed performance differences between the two datasets.

However, upon closer examination of the error map, it becomes apparent that, in the specular regions, the errors are more pronounced compared to other areas for both tested methods. The spectral reconstruction encounters notable challenges in accurately capturing and reproducing these specular reflections, leading to an increased error in these specific regions. In the context of the Sample Painting (Figure 4, third row), the white pixels stand out significantly in error, particularly in regions with saturated appearance. These pronounced errors are closely linked to the over-exposition of certain regions in the images, which poses a significant challenge for the neural-based spectral reconstruction model in accurately representing these regions. The struggle to reconstruct these over-exposed areas correctly contributes to the observed increase in errors. Additionally, it is worth noting that Y.T. Lin et al. [45] demonstrated that under-exposure spectral images similarly affects the neural based model performance, emphasising the sensitivity of the spectral reconstruction process to both over- and under-exposed conditions.

**Figure 5:**
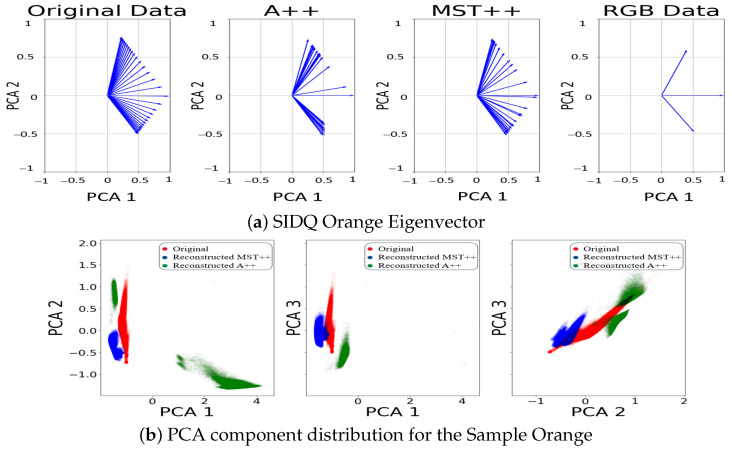
(**a**) Comparison of the eigenvectors of the first two components from the PCA. (**b**) PCA distribution between the original spectral image (red), MST++-reconstructed spectral image (blue), and A++-reconstructed spectral image (green), while the black area is a combination of the distributions for sample Orange.

**Figure 6:**
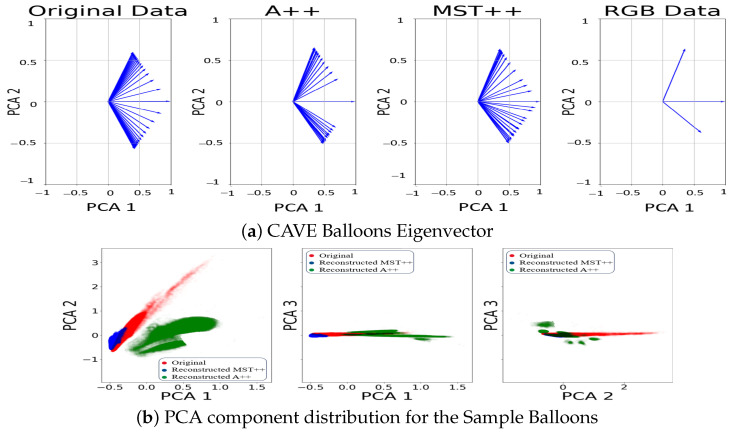
(**a**) Comparison of the eigenvectors of the two first components from the PCA. (**b**) PCA distribution between original spectral image (red), MST++-reconstructed spectral image (blue), and A++-reconstructed spectral image (green), while the black area is a combination of the distributions for sample Balloons.

**Figure 7:**
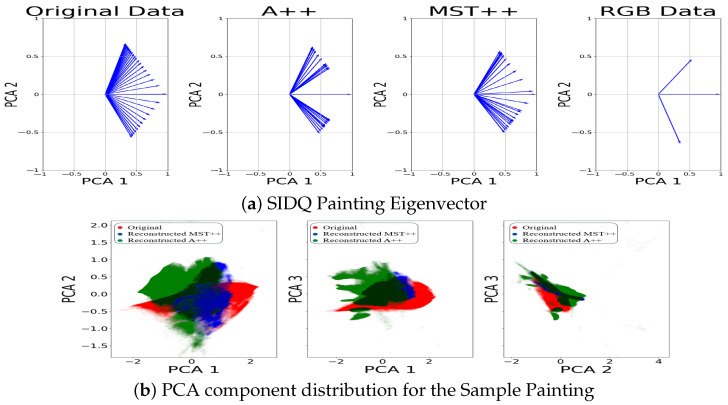
(**a**) Comparison of the eigenvectors of the two first components from the PCA. (**b**) PCA distribution between original spectral image (red), MST++-reconstructed spectral image (blue), and A++-reconstructed spectral image (green), while the black area is a combination of the distributions for sample Painting.

Furthermore, our investigation (see Figure 5, Figure 6 and Figure 7, first rows) into the spectral information contained in the reconstructed and original spectral images has brought to light discernible disparities between the two spectral images. The reconstructed data for both models, notably, may not faithfully replicate the exact spectral information inherent in the original spectral image. The challenges encountered in accurately representing over-exposed areas, among other factors, highlight a fundamental limitation: spectral reconstruction does not capture the full extent of the spectral information. This underscores the necessity for prudence in interpreting the spectral content of the reconstructed data.

Moreover, to delve deeper into the distribution of data, we extended our analysis by examining the eigenvectors of each of the two first principal components obtained from the PCA for the reconstructed spectral data, original spectra, and RGB counterpart. The eigenvectors represent the direction of maximum variance within the data. Plotting these eigenvectors enables a direct comparison between reconstructed spectral data, original spectral data and the RGB data. In Figure 5, Figure 6 and Figure 7, second row, we observe that the eigenvectors of the reconstructed spectral image occupy an intermediate position between the RGB eigenvectors and those derived from the original spectral image. This suggests that the reconstructed data capture some, but not all, of the spectral variability present in the original data. The alignment of the reconstructed eigenvectors with the RGB eigenvalues highlights a partial convergence of information between the colour channels and the reconstructed spectral space.

### 4.2. Colorimetric Analysis

In our analysis of the spectral reconstruction results (see Table 3 and Table 4), we start by looking at the average Euclidean distance values across all scenes under different lighting (D65, A, LED-B1). Table 3 presents the Euclidean distance ($\Delta E_{ab}^{*}$) between the original spectral data and the reconstructed spectra for two distinct models (A++ and MST++), under three different illuminants (D65, A, and B1), across all scenes from the SIDQ dataset. Moreover, Table 4 also shows the Euclidean distance ($\Delta E_{ab}^{*}$) between the original spectral data and the reconstructed spectra for the two models (A++ and MST++), evaluated under three different illuminants (D65, A, and B1) for all scenes from the CAVE database.

**Figure 8:**
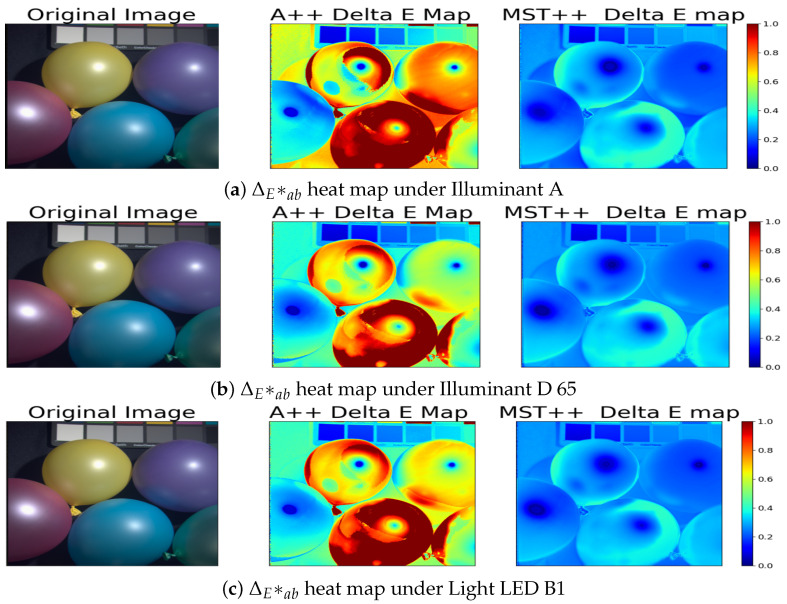
Sample Balloons from the CAVE dataset. (**Left**): Original RGB image. (**Middle**): Delta E Map from the A++ reconstruction model. (**Right**): Delta E Map from the MST++ reconstruction model.

**Figure 9:**
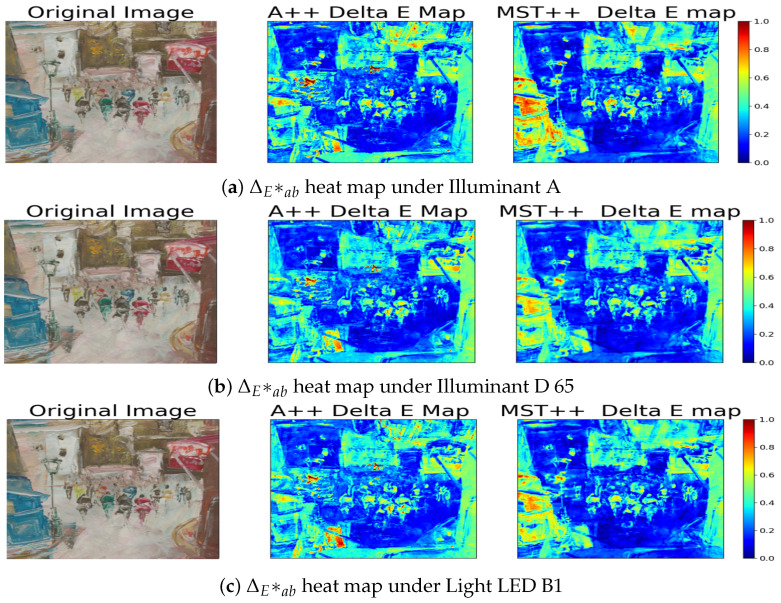
Sample Painting from the SIDQ dataset. (**Left**): Original RGB image. (**Middle**): Delta E Map from the A++ reconstruction model. (**Right**): Delta E Map from the MST++ reconstruction model.

Surprisingly, these values consistently stay below 1, showing a strong match between predicted and actual spectral data across various lighting conditions [52]. Moreover, the MST++ spectral reconstruction model stands out for its notably better performance in comparison to the A++ model. This indicates its proficiency in accurately reproducing colours even under different illuminants, which significantly impacts image quality. Consistent with our spectral analysis, the heat maps in Figure 8 and Figure 9 corroborate our previous findings that dark and specular regions have lower performance in terms of colour accuracy. This is evident from the higher $\Delta E_{ab}^{*}$ values observed in these regions compared to other areas in the scenes.

## 5. Conclusions

In conclusion, spectral reconstruction from RGB imagery holds promise for revolutionising computer vision tasks by providing access to rich and extensive spectral data without the need for expensive and complex data-acquisition campaigns. The adoption of state-of-the-art models, coupled with comprehensive datasets, can yield accurate and effective spectral reconstruction. However, challenges persist, primarily concerning reconstruction errors associated with over- and under-exposed areas, as well as the fidelity of the reconstructed information. While common performance metrics suggest good results, a closer look at the spectral distribution of the reconstructed data reveals some areas for improvement.

The colorimetric analysis of relighting from spectra further emphasises the robustness of spectral reconstruction techniques, particularly the MST++ method, in faithfully reproducing colours across different illuminants. This underscores the potential for enhancing image quality and colour fidelity in practical applications.

Therefore, methods should undergo rigorous testing against real spectral data to validate their applicability in practical settings. Furthermore, an avenue for future research lies in training deep learning models using spectral reconstructed data to investigate their behaviour and potential for achieving superior results compared to using actual spectral data. Future works may also consider the development of quality metrics for RGB-to-spectral methods based on our observations. This article underscores the promising prospect of employing spectral data, rather than RGB data, across diverse computer vision applications.

## Figures and Tables

**Table 1:** Performance-based metrics between the original spectral data and the reconstructed spectral data for the SIDQ dataset. Arrows indicate the performance trend: ↑ denotes that higher values are better, and ↓ denotes that lower values are better.

Scene	PSNR ↑		SSIM ↑		MRAE ↓		ES ↑	
	A++	MST++	A++	MST++	A++	MST++	A++	MST++
Cork	33.46	38.21	0.9907	0.9982	0.153	0.063	0.967	0.952
Hat	27.03	30.13	0.9829	0.9954	0.234	0.109	0.899	0.962
Leaves	36.34	37.96	0.9932	0.9946	0.132	0.076	0.988	0.915
Orange	30.38	34.27	0.9367	0.9971	0.266	0.090	0.937	0.931
Painting	34.36	36.54	0.9919	0.9979	0.094	0.075	0.969	0.939
Paper 1	22.37	28.42	0.9658	0.9836	0.230	0.163	0.950	0.974
Skin 1	31.63	40.95	0.9881	0.9987	0.197	0.061	0.939	0.984
Skin 2	25.84	29.17	0.9877	0.9939	0.202	0.123	0.943	0.917
Wood	35.93	39.31	0.9947	0.9984	0.1313	0.076	0.937	0.864
Average	30.70	34.65	0.9831	0.9953	0.182	0.092	0.943	0.936

**Table 2:** Performance-based metrics between the original spectral data and the reconstructed spectral data for the CAVE dataset. Arrows indicate the performance trend: ↑ denotes that higher values are better, and ↓ denotes that lower values are better.

Scene	PSNR ↑		SSIM ↑		MRAE ↓		ES ↑	
	A++	MST++	A++	MST++	A++	MST++	A++	MST++
Balloons	24.89	26.10	0.9674	0.9927	0.4119	0.146	0.902	0.913
Beads	25.07	28.76	0.9855	0.9952	0.2776	0.0968	0.9457	0.7791
CD	28.49	35.15	0.946	0.997	0.575	0.072	0.9373	0.7997
Chart	22.89	27.26	0.9674	0.9806	0.415	0.227	0.9561	0.8665
Clay	29.94	33.46	0.9850	0.9968	0.294	0.063	0.8992	0.8949
Cloth	25.85	29.98	0.9771	0.9960	0.3364	0.1208	0.9307	0.8461
Egyptian Statue	26.16	28.18	0.9676	0.9942	0.4478	0.1144	0.8114	0.6485
Face	21.66	24.59	0.9845	0.9913	0.2943	0.1648	0.8806	0.7649
Beers	25.86	28.75	0.9908	0.9883	0.1749	0.1996	0.8050	0.9753
Food	29.09	32.00	0.9906	0.9963	0.2297	0.0854	0.8676	0.8477
Lemon Slices	31.85	34.14	0.9915	0.9971	0.2195	0.092	0.8336	0.8066
Lemon	24.36	27.79	0.9909	0.9939	0.2224	0.1319	0.8643	0.7872
Peppers	26.31	28.76	0.9909	0.9945	0.2231	0.1119	0.9549	0.8100
Strawberries	29.65	31.59	0.9620	0.9961	0.4762	0.1035	0.8961	0.7992
Sushi	34.70	39.97	0.9756	0.9985	0.3892	0.0465	0.8892	0.8674
Tomatoes	35.09	39.25	0.9708	0.9984	0.4267	0.0472	0.8034	0.8144
Feathers	22.24	26.31	0.9800	0.9930	0.3276	0.1273	0.8974	0.7680
Flowers	22.93	25.62	0.9632	0.9924	0.4645	0.1375	0.8779	0.7484
Glass Tiles	26.48	28.84	0.9864	0.9950	0.2685	0.1153	0.9486	0.7792
Hairs	24.24	25.04	0.9909	0.99179	0.2167	0.1490	0.8960	0.8563
Jelly Beans	24.42	25.21	0.9864	0.9925	0.2645	0.1492	0.9040	0.7114
Oil Painting	25.08	27.05	0.9920	0.9935	0.2004	0.1420	0.9532	0.9113
Paints	21.26	22.23	0.9617	0.9889	0.4419	0.1780	0.9485	0.8869
Photo and Face	20.48	25.73	0.9858	0.9923	0.2865	0.1497	0.9125	0.7178
Pompoms	23.95	25.18	0.9600	0.9919	0.4554	0.1491	0.9575	0.8082
Apples	29.88	31.28	0.9639	0.9959	0.4711	0.0973	0.8320	0.7155
Peppers	21.66	23.93	0.9759	0.9906	0.3579	0.1679	0.9196	0.7983
Sponges	21.63	22.43	0.9635	0.9828	0.4141	0.1967	0.9675	0.8429
Stuffed Toys	25.08	26.87	0.9743	0.9933	0.3747	0.1237	0.9291	0.8464
Superballs	33.04	34.96	0.9791	0.9973	0.3532	0.0596	0.8943	0.8665
Thread Spools	25.19	28.28	0.9885	0.9942	0.2506	0.1273	0.8529	0.7596
Average	26.45	29.12	0.9826	0.9901	0.3521	0.1298	0.9289	0.8874

**Table 3:** $\Delta E_{ab}^{*}$ difference between the color images computed from the reconstructed data and the original data for SIDQ dataset for the considered lights.

Scene	CIE D65		CIE A		LED B1	
	A++	MST++	A++	MST++	A++	MST++
Cork	0.440	0.203	0.455	0.229	0.484	0.2
Hat	0.650	0.764	0.795	0.604	0.950	0.703
Leaves	0.503	0.310	0.578	0.365	0.523	0.346
Orange	0.306	0.654	0.569	0.462	0.481	0.599
Painting	0.287	0.293	0.301	0.305	0.310	0.287
Paper 1	0.592	0.547	0.785	0.497	0.599	0.516
Skin 1	0.385	0.240	0.812	0.254	0.874	0.226
Skin 2	0.270	0.217	0.737	0.256	0.721	0.217
Wood	0.370	0.299	0.683	0.334	0.614	0.303
Average	0.422	0.391	0.635	0.367	0.617	0.377

**Table 4:** $\Delta E_{ab}^{*}$ difference between the color images computed from the reconstructed data and the original data for the CAVE dataset for the considered lights.

Scene	CIE D65		CIE A		LED B1	
	A++	MST++	A++	MST++	A++	MST++
Balloons	0.58	0.27	0.680	0.27	0.633	0.263
Beads	0.73	0.33	0.907	0.29	0.760	0.334
CD	0.46	0.22	0.603	0.26	0.449	0.247
Chart	0.32	0.21	0.419	0.24	0.354	0.229
Clay	0.68	0.28	0.929	0.25	0.731	0.279
Cloth	0.44	0.24	0.392	0.21	0.481	0.253
Egyptian Statue	0.31	0.21	0.422	0.23	0.329	0.204
Face	0.32	0.25	0.426	0.26	0.368	0.246
Beers	0.31	0.21	0.361	0.21	0.355	0.205
Food	0.54	0.23	0.677	0.19	0.581	0.215
Lemon Slices	0.37	0.25	0.472	0.26	0.395	0.253
Lemon	0.52	0.28	0.804	0.27	0.523	0.285
Peppers	0.63	0.27	0.835	0.275	0.679	0.287
Strawberries	0.43	0.29	0.541	0.271	0.456	0.268
Sushi	0.37	0.19	0.515	0.214	0.407	0.217
Tomatoes	0.36	0.21	0.506	0.201	0.390	0.205
Feathers	0.52	0.27	0.720	0.225	0.569	0.249
Flowers	0.43	0.27	0.583	0.245	0.501	0.249
Glass Tiles	0.60	0.22	0.713	0.237	0.627	0.247
Hairs	0.33	0.22	0.378	0.236	0.311	0.221
Jelly Beans	0.51	0.26	0.571	0.264	0.521	0.261
Oil Painting	0.47	0.26	0.601	0.261	0.474	0.259
Paints	0.39	0.20	0.498	0.212	0.445	0.212
Photo and Face	0.29	0.26	0.398	0.274	0.327	0.255
Pompoms	0.65	0.29	0.845	0.209	0.744	0.236
Apples	0.45	0.23	0.580	0.242	0.492	0.249
Peppers	0.60	0.30	0.999	0.287	0.669	0.306
Sponges	0.81	0.29	0.130	0.240	0.925	0.265
Stuffed Toys	0.45	0.24	0.545	0.202	0.474	0.242
Superballs	0.53	0.28	0.660	0.243	0.531	0.272
Thread Spools	0.41	0.24	0.573	0.271	0.413	0.275
Average	0.477	0.259	0.589	0.243	0.513	0.251

## Data Availability

The raw data supporting the conclusions of this article will be made available by the authors on request.
